# Editorial: Occupational and environmental health in middle-aged and older adults

**DOI:** 10.3389/fpubh.2023.1196186

**Published:** 2023-05-04

**Authors:** Zhaomin Chen, Wenzhen Li, Yansen Bai, Yufeng Chen, Sheikh M. Alif, Dongming Wang

**Affiliations:** ^1^Department of Occupational and Environmental Health, School of Public Health, Tongji Medical College, Huazhong University of Science and Technology, Wuhan, China; ^2^Key Laboratory of Environment and Health, Ministry of Education & Ministry of Environmental Protection, and State Key Laboratory of Environmental Health (Incubating), School of Public Health, Tongji Medical College, Huazhong University of Science and Technology, Wuhan, China; ^3^Jockey Club School of Public Health and Primary Care, The Chinese University of Hong Kong, Hong Kong, China; ^4^School of Public Health, Guangzhou Medical University, Guangzhou, China; ^5^Unit of Integrative Epidemiology, Institute of Environmental Medicine, Karolinska Institute, Stockholm, Sweden; ^6^School of Public Health and Preventive Medicine, Monash University, Melbourne, VIC, Australia; ^7^School of Population and Global Health, The University of Melbourne, Melbourne, VIC, Australia

**Keywords:** occupational and environment health, middle-aged and older adults, occupational hazards, environmental contaminants, adverse effects

A healthy workplace and conducive living environment are fundamental for the health, wellbeing, and progress of modern society. However, rapid industrialization, rising living demands, and the intensification of globalization have all led to increased occupational hazards and contamination of the air, water, soil, and food, thus posing new challenges for occupational and environmental health. Multiple organs and systems of the human body might suffer short- or long-term adverse effects from occupational hazards and environmental contaminants. For example, particulate matter, one of the primary contributors of air pollution, can harm the respiratory and cardiovascular systems by inducing oxidative stress, inflammation, and activation of the autonomic nervous system (1, 2). Meanwhile, there are too many diverse workplace hazards to exhaust their negative consequences and underlying mechanisms; hence, complex multidisciplinary study approaches are warranted.

Extended and recurrent occupational and environmental exposure can amplify these adverse effects (3). The threat of occupational noise exposure to the cardiovascular system rises with increasing cumulative noise exposure or duration of exposure time (4, 5). Thus, more attention should be given to middle-aged and older adults, who frequently have increased health risks and prolonged exposure to occupational and environmental hazards.

Currently, emerging new technologies and instruments have made it feasible to investigate the underlying mechanisms behind occupational and environmental hazards and their adverse health outcomes. The multi-omics approach, integrating data from multiple levels (genes, mRNAs, regulatory factors, proteins, metabolism, etc.), provides a greater knowledge of the molecular mechanisms and genetic foundation of complex features in biological processes and disease processes (6).

With this Research Topic on “occupational and environmental health in middle-aged and older adults,” we aimed to entice more academics to concentrate on the health effects of occupational and environmental hazards along with their underlying potential causal mechanisms. Moreover, we aimed to motivate more scholars to explore early biomarkers of environmental and occupational hazards for both exposure and harmful health impacts. This will offer insights for enhancing hazards prevention and control.

Concerns regarding occupational and environmental health threats are growing. The majority of the submitted manuscripts investigated the adverse effects of hazards on the general population (Ba̧czalska et al.; Xia et al.), specific occupational groups (Chen et al.), or middle-aged and older adults (Li et al.; Wang et al.; Huang et al.; Ren et al.; Shen et al.; Wei et al.); additionally, several studies proposed innovative approaches to identify the hazards (Zhang et al.).

Indoor air pollution from the burning of household solid fuel has been found to be significantly associated with cognitive decline, visual impairment, and depression, which are risk factors for functional disability (FD). Given that older adults typically spend the majority of their time indoors, particularly after retirement age, their health is more likely to be impaired by prolonged exposure to indoor air pollution. In a cohort study with 17,708 participants aged 45 years and older from 450 villages/urban communities across China, Ren et al. identified that household solid fuel use was a risk factor for FD, and switching from solid to clean household fuel could help to reduce the burden of FD in the currently aging society of China.

Volatile organic compounds (VOCs) are a large group of chemicals widely used in people's daily routines. Increasing evidence has revealed the VOCs' accumulating toxicity. In a cross-sectional study based on the United States National Health and Nutrition Examination Survey database, Wei et al. explored the relationship between blood VOCs and a prostate-specific antigen and found a positive association between blood chloroform and the total prostate-specific antigen level.

Telomeres are DNA protein complexes that protect the ends of eukaryotic chromosomes, which shorten each time a cell is divided. Telomere shortening is not only a key mechanism of cell senescence, but it also contributes to aging and diseases.

Xia et al. screened the possible hazardous urinary metals for leukocytes telomere length (LTL) while constructing an artificial neural network model to make the prediction of LTL based on urinary metals, demography, behavior, and disease history.

The prevalence of mild cognitive impairment (MCI), which is the most common early sign of Alzheimer's disease, increases with age. The findings of the study by Wang et al. based on participants aged 60 years and older, imply that exposure to chemical agents such as cesium, manganese, barium, and cadmium may be involved in the pathophysiology of MCI, such as via interfering with potassium channels or protecting neurons.

Noise is defined as an unpleasant or harmful sound. Aircraft noise is characterized as the highest source of annoyance when compared with other environmental noise sources, as addressed by Ba̧czalska et al. who provided an updated literature review on aircraft noise exposure and the consequences for cardiovascular systems in the context of the World Health Organization Environmental Noise Guidelines for the European Region (for the whole of Europe).

Non-Gaussian complex noise is composed of transient high-energy impulsive noise superimposed on Gaussian background noise, which is common in the occupational environment and could lead to more severe hearing damage than steady noise. Conventional noise measurement techniques are not suitable for complex noise measurement due to the peak clipping effect of impulse noise. Thus, based on previous studies and literature reviews, Zhang et al. introduced a draft guideline for occupational workplaces in China to measure workplace non-Gaussian complex noise exposure using kurtosis adjustment of the noise level.

Through a survey of enterprise workers in key industries in China, Chen et al. investigated the prevalence of musculoskeletal diseases of the wrists among occupational workers in different industries. They identified several factors (e.g., female sex, working age, and poor wrist posture) that were associated with wrist injuries and provided a scientific basis for formulating corresponding measures for improving occupational workers' health.

Humans are unavoidably exposed to a range of occupational or environmental hazards every day. Sometimes, even slight modifications to the physical or chemical properties of certain dangerous substances can have a profound effect on human health. Through this Research Topic, we have sought to increase attention and interest in strengthening the potential links between occupational and environmental hazards and population health, from exploring their relationships to investigating potential mechanisms and biomarkers. It provides more knowledge to assist with the development of public health policies, and it also improves tertiary prevention measures and targeted prevention for populations that are especially exposed. Population aging is now a significant global public health concern. From a broader perspective, concentrating on the health hazards faced by older adults can assist in enhancing their quality of life and lessening the burden of disease.

## Author contributions

All authors listed have made a substantial, direct, and intellectual contribution to the work and approved it for publication.
